# Virus-induced gene silencing and virus-induced flowering in strawberry (*Fragaria* × *ananassa*) using apple latent spherical virus vectors

**DOI:** 10.1038/s41438-018-0106-2

**Published:** 2019-02-01

**Authors:** Chunjiang Li, Noriko Yamagishi, Ichiro Kasajima, Nobuyuki Yoshikawa

**Affiliations:** 1 Faculty of Agriculture, Iwate University, Morioka 3-18-8, Iwate, 020-8550 Japan; 2 Agri-Innovation Research Center, Iwate University, Morioka 3-18-8, Iwate, 020-8550 Japan

**Keywords:** Agricultural genetics, Plant molecular biology

## Abstract

Apple latent spherical virus (ALSV) vector is a convenient alternative to genetic transformation in horticultural plants, especially in species recalcitrant to genetic transformation. ALSV, an RNA virus, can infect a wide variety of plant species including major horticultural plants without inducing symptoms. Here, methodologies were developed for infection of ALSV vectors to strawberry seedlings and plantlets cultured in vitro. A seed-propagated F_1_ hybrid strawberry cultivar 'Yotsuboshi' was aseptically grown on half-strength Murashige–Skoog medium for 1 month and true leaves were inoculated with an ALSV RNA preparation by particle bombardment. ALSV vector infection rates varied from 58 to 100% according to the insertion sequences, in ‘Yotsuboshi’ seedlings. Plantlets (‘Dover’) propagated in vitro could also be infected with ALSV vector at a similar infection rate. For virus-induced gene silencing (VIGS), we prepared an ALSV vector carrying a 201 nucleotide segment of the strawberry *phytoene desaturase* gene. ‘Yotsuboshi’ and ‘Dover’ plants infected by this vector generated completely white leaves at fifth or sixth true leaves and above. For virus-induced flowering (VIF), we used an ALSV vector expressing the *Arabidopsis thaliana flowering locus T* gene. Strawberry seedlings infected by this vector started to flower from about 2 months post inoculation and bore fruits with viable seeds. The ALSV vector was no longer detected in any of the seedlings from early-flowered strawberries. Thus, the ALSV vector may be beneficial for examination of gene functions by VIGS in strawberry, and VIF using ALSV vector constitutes an effective new plant breeding technique for the promotion of cross-breeding in strawberry.

## Viral vectors: Easy gene modification in strawberries

Apple latent spherical virus (ALSV) offers a powerful tool to reveal strawberry gene function and induce early flowering. Preferential strawberry traits are highly influenced by their genetics; however, the specific mutations that change these traits are largely unknown. This prompted Nobuyuki Yoshikawa and his team from Iwate University, Japan, to develop ALSV to infect and introduce intended genetic material into strawberries. Infecting *in vitro* cultured plants at a rate of 58—100%, ALSV-vectored gene introduction proved able to ‘silence’ a natural strawberry gene, constituting a method to identify gene functions. The infection was not pathogenic and did not carry on to the plants’ progeny. The team also used ALSV to introduce a gene that induced flowering 2 months post inoculation, a technique that could be used in future to promote shorter cross-breeding times.

## Introduction

Strawberry (*Fragaria* × *ananassa*) is a popular crop worldwide with production exceeding four million tons per year^1^. Despite the long cultivation history of strawberries from the fiteenth century, strawberry breeding remains a major concern of farmers and researchers with regard to a variety of traits such as fruit quality (taste), fruit colour, fruit firmness, fruit storability, ascorbic acid content, everblooming habit, disease resistance and heat/cold resistance^2^. Changes in these agronomically important traits are expected to be caused by mutations in genomic DNA. For example, the everblooming (continuous flowering) trait of woodland strawberry (*Fragaria vesca*) is caused by a 2 bp deletion in the coding region of the *KSN* gene^3^, a strawberry homologue of the *Terminal Flower 1* (*TFL1*) gene. The genetic mapping approach has also identified the *FaOMT* gene mutation as the regulator of variation in the release of mesifurane, one of the volatiles of strawberry fruit, based on complete co-segregation of the identified 30-bp mutation in the *FaOMT* promoter^4^.

Specific genetic mutations causing changes in other agronomically important traits in strawberry remain mostly unknown, although candidate genes are being suggested through experimental efforts such as gene expression analyses^5–8^. The release of genomic sequences and reports of thousands of DNA markers to detect polymorphisms on the chromosomes are also expected to greatly accelerate both forward and reverse genetic studies of strawberry^9^. Such knowledge with regard to agronomically important genes and their mutations both informs basic plant biology and accelerates strawberry breeding based on DNA information^10^.

Following identification of agronomically important or candidate genes, their characterization by genetic overexpression or suppression represents a standard strategy for confirmation of their functions. The protocol for genetic transformation of strawberry by *Agrobacterium tumefaciens* was first established in 1990^11,12^. Whereas the transformation efficiency is generally around 5%, the efficiency may be increased up to 100%^13–16^, although transformation rate appears to depend on the strawberry cultivar. Specifically, 100% transformation efficiency was achieved in an everbearing (day-neutral) cultivar ‘Calypso’, using the super-virulent *Agrobacterium* strain AGL0^14^.

Transient expression/suppression by infiltration of *Agrobacterium* into strawberry fruits has also been utilized for rapid analysis of gene functions. In this case, *Agrobacterium* causes overexpression or RNA interference of the target gene^17,18^, depending on the nucleotide sequence introduced into the transfer DNA region of the binary plasmid. The target strawberry gene may also be suppressed via virus-induced gene silencing (VIGS) using tobacco rattle virus (TRV) vectors^19,20^. Considering that genetic transformation typically requires 15 months from the start of the experiments to the production of strawberry fruits^18^, these transient systems enable much faster estimation of gene function than stable transformation, although the genes are expressed/suppressed only in sections within strawberry fruits (more strictly, receptacles). Alternatively, genes can be expressed/suppressed in whole strawberry fruits when *Agrobacterium* is repeatedly injected into fruits at least three times^18^. Such transient systems are currently utilized for the analysis of gene functions with regard to strawberry fruit phenotypes, such as pigmentation, aroma generation, ripening and disease resistance^21^. Genes can also be transiently expressed in strawberry leaves by infiltration of *Agrobacterium* for the analysis of their functions^22^.

Once agronomically important mutations in the genome are identified, they can be combined by cross-breeding and DNA marker selection. However, the long generation time of crops often constitutes a major problem for efficient cross-breeding. Although the genomes of vegetatively propagated cultivars/crops such as apple and pear remain genetically heterozygous, short generation time is also important when the crop genome is homogenized for establishment of seed-propagated genetically homozygous cultivars/crops and F_1_ hybrid cultivars/crops, such as rice and maize. Thus, in addition to controlling growth conditions such as cultivation in greenhouse or incubator, high CO_2_ levels, tiller removal, paclobutrazol treatment, grafting on rootstock, and embryo rescue^23–26^, transgenic expression of the *leafy* or *flowering locus T* (*FT*) gene or suppression of the *TFL1* gene also comprise important techniques for inducing early flowering and reducing generation time^25,27,28^.

Virus-induced flowering (VIF) can also be effective for reducing the generation time of crops. In VIF, crops are infected with RNA virus vectors expressing an *FT* gene to induce early flowering^29^. An advantage of VIF is that the genomic DNA of crops is not transformed, and the infected transgenic virus is rarely carried to the progeny (next-generation) plants. In addition, virus infection does not depend on the specific crop cultivar in many cases. Rather, infectivity of virus vectors depends on the host range of viruses; thus, zucchini yellow mosaic virus has been used for the expression of *Arabidopsis thaliana FT* (*AtFT*) in squash/pumpkin (*Cucurbita moschata*)^30^, cotton leaf crumple virus was used for *AtFT* expression in cotton^31^ and citrus leaf blotch virus was used for *AtFT* or citrus *FT* expression in citrus plants^32^ for the successful induction of early flowering. As apple latent spherical virus (ALSV) exhibits a relatively wide host range, in previous studies we used an ALSV vector for the expression of *AtFT* or other *FT* genes such as a gentian *FT* to induce early flowering in *A. thaliana*, tobacco (*Nicotiana*) plants, soybean, apple, petunia, lisianthus (eustoma) and gentian^33–37^. The progeny plants of cross-bred gentian generated by using ALSV-based VIF were confirmed for elimination of the virus, cleared governmental review and started to be grown in an open field in Hachimantai City of Japan for selection of a new cultivar. This appears to be the first example of the application of any New Plant Breeding Technique in Japan.

Again, ALSV enjoys advantages as a virus vector: ALSV has a wide host range, latently infects most plant species (without viral symptoms) and evenly infects the upper leaves. ALSV may provide novel high-throughput methods for estimation of the functions of strawberry genes, and promotion of cross-breeding by shortening the generation time of strawberry. To examine the utility of this technique, in the present study we inoculated ALSV vectors to a commercially important cultivar of strawberry. As a result, the abilities of the ALSV vector to successfully infect strawberry seedlings and to spread to every organ of the shoot, as well as to induce VIGS and VIF in strawberry plants, were ascertained.

## Results

### Conditions of ALSV inoculation

**Fig. 1:**
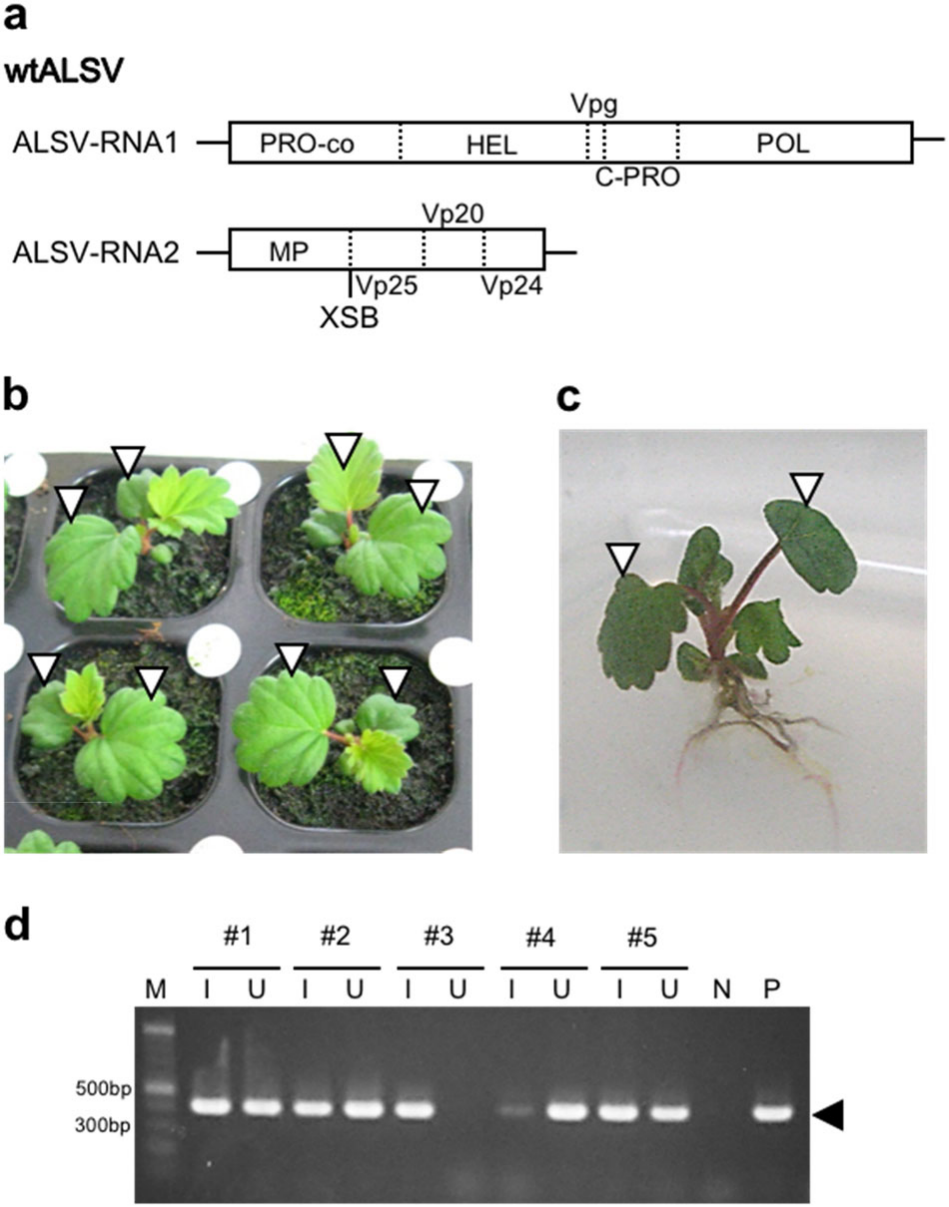
Inoculation of wtALSV to strawberry seedlings. **a** Structure of the wtALSV. RNA1 genome encoding protease cofactor (PRO-co), NTP-binding helicase (HEL), viral protein genome-linked (Vpg), cysteine protease (C-PRO) and RNA polymerase (POL). The RNA2 genome encodes movement protein (MP), and capsid proteins Vp25, Vp20 and Vp24. An *Xho*I–*Sma*I–*Bam*HI (XSB) cloning site was inserted in the plasmid clone of the RNA2 genome, between MP and Vp25. **b** Strawberry seedlings grown on soil in a plug tray. **c** Strawberry seedling aseptically grown on solid medium. Open arrowheads in **b** and **c** indicate expanded true leaves in which gold particles were bombarded. **d** RT-PCR assay of ALSV infection to strawberry. #1 through #5 indicate independent strawberry seedlings grown in vitro and inoculated with wtALSV. Symbols, I and U, indicate inoculated leaves and uninoculated upper leaves, respectively. Non-inoculated strawberry plant (N) and wtALSV-infected *Chenopodium quinoa* plant (P) were also analysed as controls

As ALSV vectors have not yet been tested in strawberry, we first aimed to identify a successful condition of ALSV inoculation to strawberry. ALSV consists of RNA1 and RNA2 genomes, with each genome encoding a single polyprotein. For comparison of inoculation conditions, ALSV vector without any insertion of external sequence (wild-type; wtALSV) was used (Fig. 1a). Because ALSV does not necessarily infect horticultural plants easily, ALSV is usually inoculated as viral RNA instead of via rub inoculation or agroinfiltration. For example, gold particles coated with viral RNA are bombarded by gene guns to cotyledons of germinating seeds in the case of apple^33,36^, or to true leaves of juvenile seedlings in the case of gentian^35,38^.

In the present study, we first attempted bombardment of RNA-coated gold particles to true leaves of strawberry seedlings (a seed-propagated cultivar ‘Yotsuboshi’) grown on soil in a plastic plug tray (Fig. 1b). As strawberries had developed three or four true leaves at this stage (1 month after sowing), gold particles were shot at two expanded true leaves. Although the amount of RNA per shot (7 μg) in this trial was greater than the standard amount (5 μg) for apple and gentian, and two shots were taken per each leaf, reverse transcription-polymerase chain reaction (RT-PCR) analysis performed on upper leaves at 1 month post inoculation revealed that none of the 16 inoculated strawberry plants was infected by wtALSV (infection rate of 0%; Table 1). Using wtALSV, we also inoculated strawberry seedlings grown aseptically on half-strength Murashige–Skoog (MS) medium with RNA-coated gold particles, under the same conditions as the plants grown in the plug tray. RT-PCR analysis was performed at 1 month post inoculation, and eight out of nine inoculated plants were infected by wtALSV (infection rate of 89%; Table 1). Fig. 1d shows the DNA bands obtained by RT-PCR analysis of strawberry seedlings grown aseptically on half-strength MS medium. The wtALSV was detected both in inoculated leaves and upper leaves of strawberry plants, except for the #3 plant in which ALSV was not detected in the upper leaf. Inoculation of wtALSV to in vitro culture of ‘Dover’ resulted in successful infection at an infection rate of 63%. Thus, seedlings and in vitro cultures grown aseptically were inoculated in the following experiments.

**Table 1:** Infection rates of ALSV vectors to strawberry seedling (‘Yotsuboshi’) and in vitro culture (‘Dover’)

Cultivar	Vector	Growth condition	Growth stage at inoculation	Infected plants/inoculated plants (%)
‘Yotsuboshi’	wtALSV	soil (plug tray)	3–4 true leaves	0/16 (0)
‘Yotsuboshi’	wtALSV	1/2 MS aseptically	3–4 true leaves	8/9 (89)
‘Yotsuboshi’	ALSV-FaPDS	1/2 MS aseptically	3–4 true leaves	12/12 (100)
‘Yotsuboshi’	ALSV-AtFT	1/2 MS aseptically	3–4 true leaves	7/12 (58)
‘Dover’	wtALSV	1/2 MS aseptically	3–4 true leaves	5/8 (63)
‘Dover’	ALSV-FaPDS	1/2 MS aseptically	3–4 true leaves	5/5 (100)

### VIGS in strawberry leaf

Next, we tested whether ALSV could be used for VIGS in strawberry leaves. In a previous report, fragmental *phytoene desaturase* (*PDS*) genes of tobacco (*tPDS*), watermelon (*cuPDS*) and soybean (*soyPDS*) were successfully used for VIGS by using ALSV vectors, and whitening of leaves (photo-bleaching) was observed in tobacco (*Nicotiana tabacum*), *Nicotiana benthamiana*, *Nicotiana glutinosa*, *Nicotiana occidentalis*, cucumber (*Cucumis sativus*), pumpkin (*Cucurbita pepo*), watermelon (*Citrullus lanatus*), luffa (*Luffa cylindrica*), bottle gourd (*Lagenaria siceraria*), soybean (*Glycine max*), pea (*Pisum sativum*), adzuki bean (*Vigna angularis*) and cowpea (*Vigna unguiculata*)^39^. The strawberry *PDS* gene (accession no. FJ795342) (designated *FaPDS*) encodes a protein of 568 amino acid residues with the protein sequence being highly homologous to tPDS, cuPDS and soyPDS, suggesting that FaPDS functions as phytoene desaturase in strawberry.

**Fig. 2:**
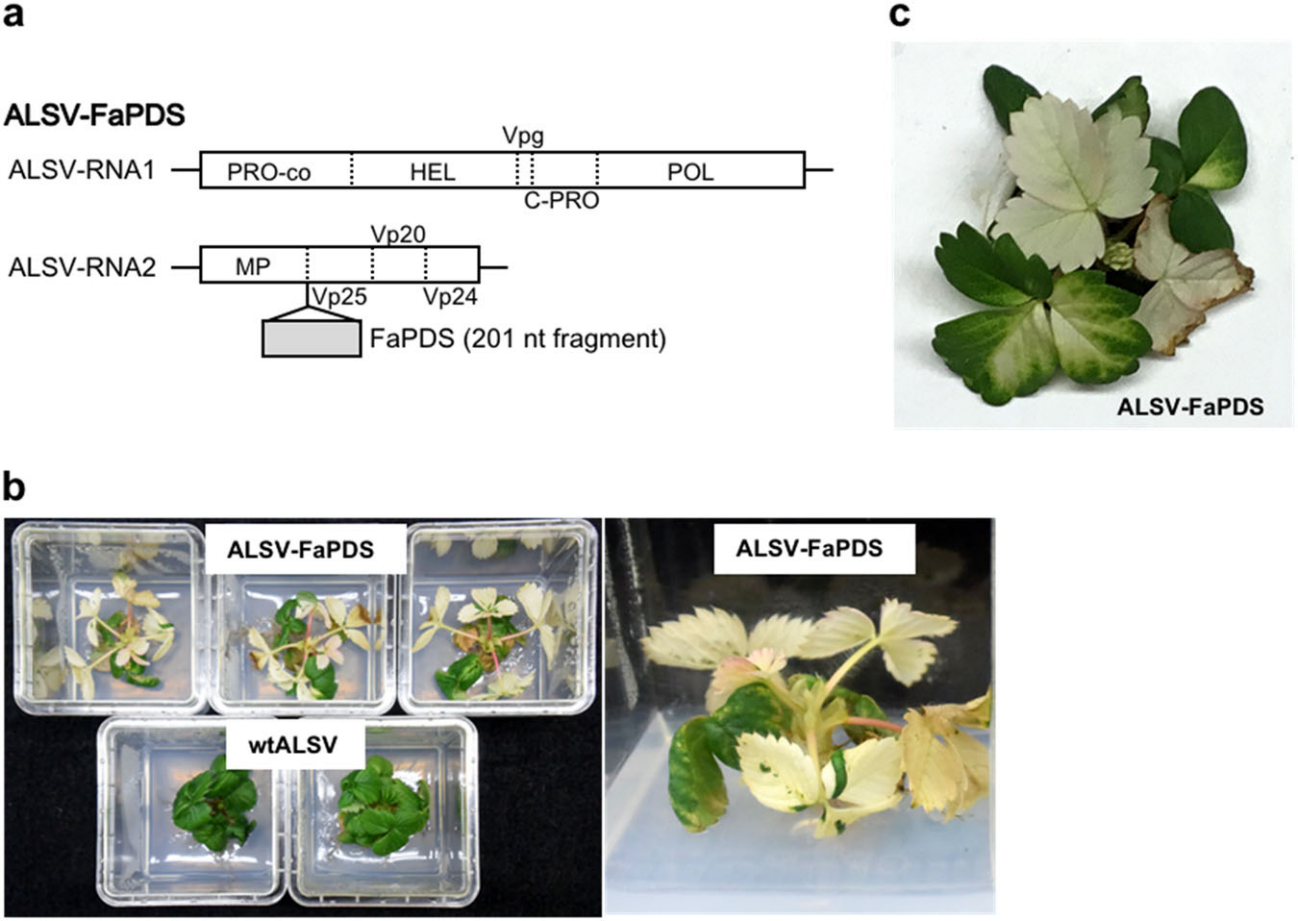
Virus-induced gene silencing in strawberry seedling (‘Yotsuboshi’) and a cultivar (‘Dover’) grown in vitro and infected with ALSV-FaPDS. **a** Structure of ALSV-FaPDS. **b** Strawberry seedlings (‘Yotsuboshi’) grown in vitro and infected with ALSV-FaPDS (left-top) or wtALSV (left-bottom). Photograph was taken at 45 days post inoculation (dpi). Right picture shows magnification of the representative plant. **c** Photo-bleaching phenotype of ‘Dover’ infected with ALSV-FaPDS (30 dpi)

Clear photo-bleaching by VIGS of *PDS* genes by ALSV vectors is almost always observed when the fragment sequences of the *PDS* genes are 200 nucleotides (nt) or longer^39^. In the present study, a 201 nt fragment of *FaPDS* was synthesized artificially and then introduced into the *Xho*I–*Sma*I–*Bam*HI (XSB) cloning site of the RNA2 vector of ALSV (Fig. 1a). Viral RNA prepared from this clone possesses an additional 201nt insertion between MP and Vp25 of the RNA2 genome (Fig. 2a). This virus was designated ALSV-FaPDS. An RNA preparation from leaves infected with ALSV-FaPDS was bombarded on true leaves of strawberry seedlings (‘Yotsuboshi’) aseptically grown on half-strength MS medium. The result indicated that all 12 strawberry plants inoculated with this virus were infected (infection rate of 100%; Table 1). Photo-bleaching was also observed in the majority of each upper leaf and leaf petiole of the plants infected by ALSV-FaPDS (Fig. 2b). Here, partial photo-bleaching was observed in ‘close’ upper leaves, which expanded immediately after inoculation, and overall photo-bleaching was observed in ‘remote’ upper leaves, which expanded later than ‘close’ upper leaves. Overall photo-bleaching of leaves was observed at least 1 month after inoculation. Conversely, photo-bleaching was never observed in the plants infected by wtALSV (Fig. 2b). A cultivar ‘Dover’ propagated from in vitro culture also showed a clear photo-bleaching phenotype of newly developed leaves after inoculation of ALSV-FaPDS (Fig. 2c). These results suggest that ALSV vector can be effectively used for the analysis of gene function of strawberry.

### VIF of strawberry

**Fig. 3:**
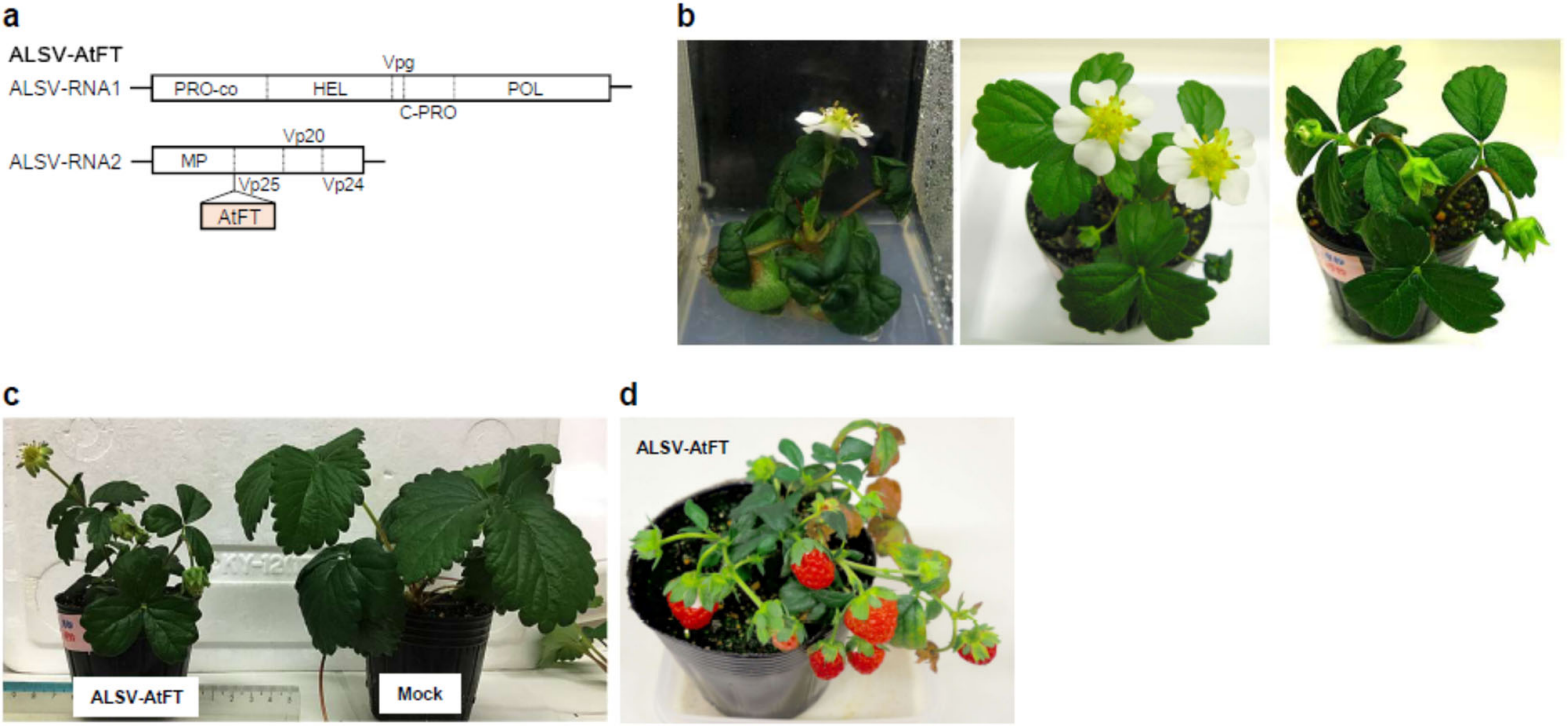
Virus-induced flowering in strawberry seedling (‘Yotsuboshi’) infected with ALSV-AtFT. **a** Structure of ALSV-AtFT. **b** Strawberry plant infected by ALSV-AtFT. Photographs were taken at 60 (left), 89 (centre), and 95 dpi (right). **c** A strawberry plant infected by ALSV-AtFT compared with a mock-inoculated control plant at 96 dpi. **d** A strawberry plant infected with ALSV-AtFT bearing fruits. Photograph was taken at 127 dpi

An ALSV vector carrying full-length AtFT inserted at the XSB site was also used in the present study. This vector, ALSV-AtFT, expresses AtFT as a part of the polyprotein encoded by the RNA2 genome of ALSV (Fig. 3a). After translation of the polyprotein, AtFT is excised from the polyprotein through digestion with protease. RNA of ALSV-AtFT was bombarded onto the true leaves of 1-month-old, aseptically grown seedlings in vitro. A total of 12 plants were inoculated in this analysis, of which seven were systematically infected by ALSV-AtFT (infection rate of 58%; Table 1). The infected plants were transferred to soil in plastic pots. Some of the infected plants set floral buds in the plant box and other plants flowered after transfer to soil in plastic pots, being self-pollinated (Fig. 3b). The size of plants infected with ALSV-AtFT, was smaller than that of mock-inoculated plants, because of the transition from the vegetative to the reproductive phase (Fig. 3c). However, fruits were mature approximately 1 month after flowering and seeds exhibited germination ability (Fig. 3d). Thus, strawberry fruits were mature 4–5 months after sowing in this analysis and seeds could be collected from mature fruits. Strawberry plants without virus infection, or plants infected by wtALSV, did not flower or bear fruit within these time frames.

### ALSV distribution in reproductive organs

**Fig. 4:**
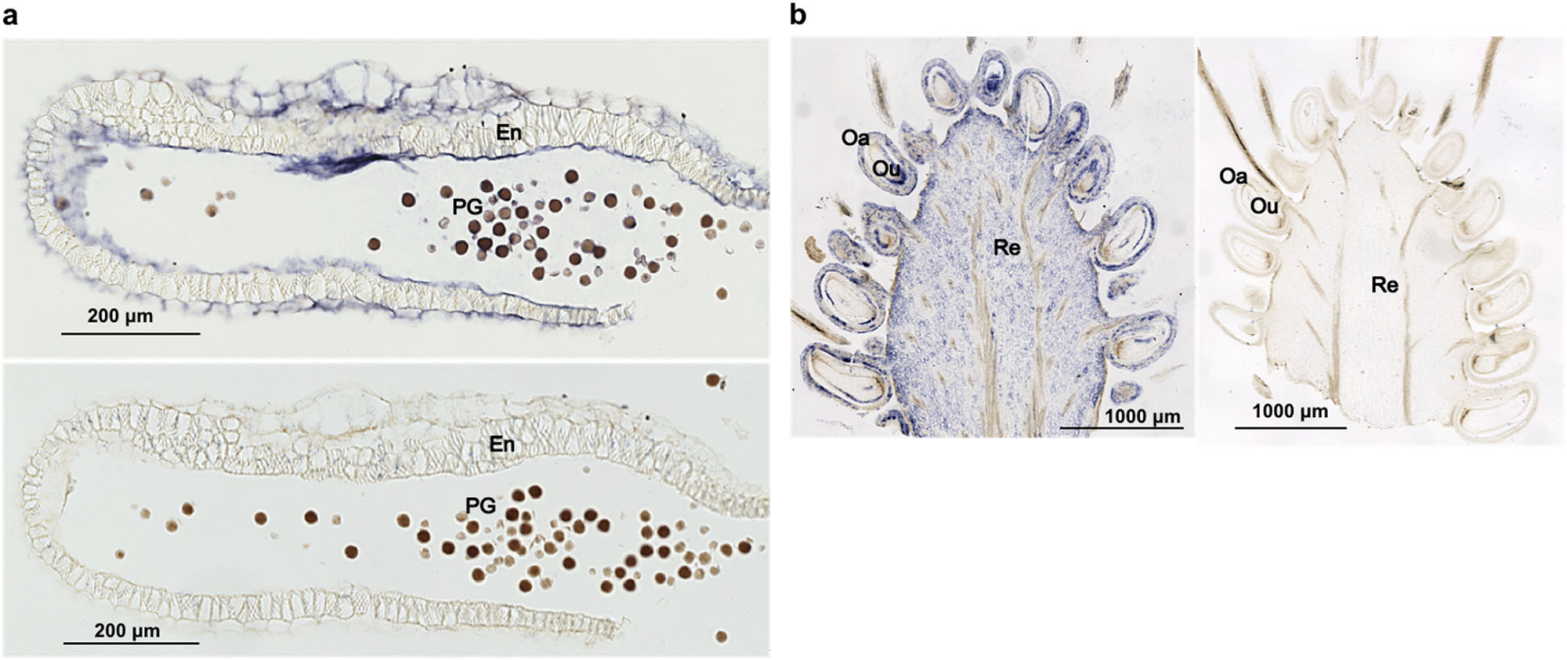
In situ hybridization analysis of ALSV-AtFT distribution. **a** ALSV distribution in the anther. ALSV was detected using an ALSV-Vp24(−) probe (top) and as a control experiment in the anther by using an SMV-P1(−) probe (bottom). Blue colour indicates distribution of ALSV in tissues. **b** ALSV distribution in fruit on plants infected with ALSV-AtFT detected by an ALSV-Vp24(−) probe (left). Control experiment in fruit using an SMV-P1(−) probe (right). PG pollen grain, En endothecium, Re receptacle, Oa ovary, Ou ovule

Observation of photo-bleaching by VIGS indicated penetration of ALSV to many of the cells in vegetative tissues, at least in the upper part of strawberry plants. ALSV distribution in reproductive organs was also estimated by microscopic observation of viral RNA following in situ hybridization analysis. Here, reproductive organs were prepared by infection of ALSV-AtFT to induce early flowering. Sections of fixed tissues were labelled with an RNA probe. The probe was further detected using an antibody, the phosphatase activity of which was used for chromogenic reaction to generate blue indigo/formazan pigmentation. When anther tissues were labelled with an ALSV probe, blue pigmentation was observed around the endothecium and in the majority of pollen grains (Fig. 4a, top). In contrast, no pigmentation was detected when anther tissues were labelled with a control SMV probe (Fig. 4a, bottom). Similarly, blue pigmentation was observed in the ovary and ovule of immature fruits using an ALSV prove (Fig. 4b, left), whereas there was no pigmentation in any part of the fruits with an SMV probe (Fig. 4b, right). Thus, ALSV was distributed in the reproductive organs (pollen grain and ovule) in strawberry flowers.

### Virus elimination in progeny plants

**Fig. 5:**
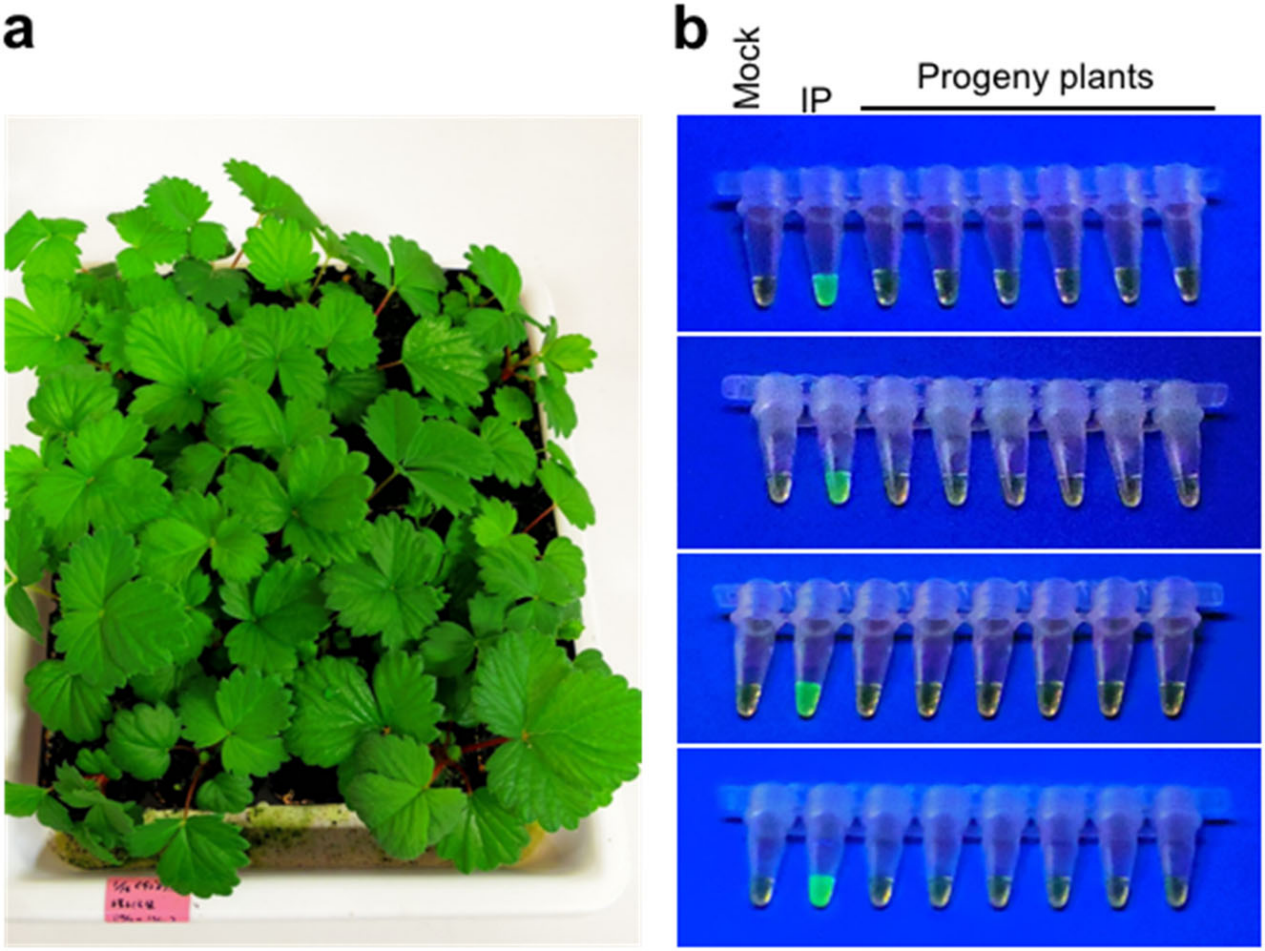
Estimation of ALSV infection in progeny plants from early-flowered plants. **a** Progeny seedlings from early-flowered strawberry through infection of ALSV-AtFT. Photograph was taken 2.5 months after sowing. **b** RT-LAMP analysis of progeny plants. 'Mock' represents reaction without template DNA/RNA. IP, infected plants.

ALSV is transmitted to progeny plants (seed transmission), at the rates of 0 to >50% depending on plant species. To examine possible ALSV transmission to progeny strawberry plants, next-generation plants were grown from strawberry seeds that were collected from early-flowered parents generated by infection of ALSV-AtFT. At 2.5 months after sowing on soil (Fig. 5a), upper leaves were tested by RT-PCR or RT loop-mediated isothermal amplification (RT-LAMP). A sum of 58 independent plants was tested by either RT-PCR or RT-LAMP, respectively, with lack of amplification signal in the former, and lack of green fluorescence of calcein in the latter (Fig. 5b), indicating the absence of ALSV in progeny plants. Thus, ALSV was not detected in any of these 116 progeny plants examined (seed transmission rate of 0%).

## Discussion

In our initial trial of ALSV inoculation to juvenile strawberry seedlings by particle bombardment, wtALSV never infected to soil-cultivated plants, whereas wtALSV infected to 89% of seedlings grown in vitro (Fig. 1; Table 1). As a similar trend of difference in infection rate was also observed in ALSV infection to grapevine (Maeda et al., unpublished data), a general difference in physiological states between soil-cultivated plants and tissue-cultured plants may exist that renders in vitro plants more susceptible to viral infection. Namai et al.^40^ reported that the resistance to anthracnose of tissue-cultured strawberry plants increased as the acclimation period increased, suggesting that the resistance of a strawberry cultivar may be induced by external factors. Considering that plants are equipped with molecular defense systems against virus infection^41^, possible environmental effects on these defense systems, such as RNA silencing mechanism, should be examined in future studies.

The infection rate of ALSV-FaPDS to seedlings grown in vitro was 100%, a similar rate as obtained with wtALSV. Conversely, infection rate of ALSV-AtFT in in vitro strawberry was only 58% (Table 1). Such variation in infection rate of ALSV has been repeatedly observed in many plant species. The infection rate of ALSV is affected (lowered) according to the size and nucleotide sequence of the insertion. However, insertions of 201 nt rarely affect infection rate. The infection rate of ALSV-AtFT is expected to be sufficiently high for basic studies and applications in strawberry. Moreover, in addition to strawberry seedling (‘Yotsuboshi’), successful infection of ALSV vectors was also achieved in strawberry cultivar (‘Dover’) propagated from in vitro culture (Table 1; Fig. 2). This suggests that ALSV technology might be applied to the analysis of gene function and promotion of flowering in the majority of strawberry cultivars.

Photo-bleaching in whole leaves, petioles and stems in the upper part of vegetative organs (Fig. 2) indicated ALSV penetration throughout the upper portions of vegetative organs. In addition, systemic infection of wtALSV did not cause viral symptoms in strawberry. These results are consistent with our previous experiments in other plant species such as apple, gentian, soybean and tobacco^35,39,42^. In addition, analysis of ALSV infection in reproductive organs (Fig. 4) demonstrated that ALSV could be detected in many parts of pollen grains and ovules, as well as in ovaries, receptacles, petals and sepals, indicating that ALSV also penetrated reproductive organs.

As described in the Introduction, TRV vector is already reported to induce gene silencing as the sections within strawberry fruits^19,20^. The advantages of TRV compared with ALSV are that the experimental procedure is quite simple, and it can be used for VIGS study in strawberry fruits. On the other hand, the advantages of ALSV would be that ALSV can be used for gene silencing in whole vegetative tissues (leaves and stems) of strawberry shoot: ALSV induces even (overall) gene silencing in stems and ‘remote’ upper leaves (Fig. 2). It is not examined yet whether ALSV is applicable to VIGS in strawberry fruits, although there will be a good chance to induce even gene silencing in vegetative tissues. What is more, ALSV can induce VIF to shorten the generation time of strawberry. In practical use of ALSV for VIF of strawberry, normally grown cultivars will be crossed with each other. The F_1_ seeds are gathered, aseptically cultured, and inoculated with ALSV to induce early flowering for estimation of fruit quality or further crossing. After elimination of ALSV, this virus will no longer affect flowering pattern or vegetative growth (plant size).

ALSV should preferably be removed in the next generation, when early flowering (VIF) technology is utilized for the promotion of cross-breeding. In this sense, VIF with ALSV vectors is especially convenient in gentian and lisianthus, in which ALSV is not transmitted to the next generation^35,43^. In the case of apple, ALSV is transferred from the seed parent to progeny plants at a low rate (0–4.5%)^44^. However, when we examined strawberry plants (Fig. 5), ALSV was never transferred to the next generation, suggesting that ALSV infecting pollen grains and ovules becomes degraded in strawberry embryos after pollination.

Shorter generation time is also advantageous for the generation of hybrid strawberry cultivars. Compared with traditional vegetative propagation using runners, seed-propagated characteristics are expected to ease the labour required for preparing new seedlings. To develop new hybrid cultivars, genomes of both parents are fixed to homozygotes through repetitive self-pollinations. After breeding of strawberry cultivars by using VIF technology, the absence of ALSV in progeny plants are ascertained by methods like RT-LAMP, before they are taken to open fields. Notably, such strawberry plants are not transgenic at least on the product base^45–47^, because ALSV, an RNA virus, only transiently infects strawberry cells. Our findings indicate that it is now possible to breed new strawberry cultivars by using ALSV-based VIF.

## Materials and methods

### Plant materials

Strawberry (*Fragaria* × *ananassa* Duchesne) cultivars ‘Yotsuboshi’ and ‘Dover’ were used in this study. ‘Yotsuboshi’ is an everbearing, seed-propagated F_1_ hybrid cultivar generated by crossing between 'Mie strain 1' (seed parent) and 'A8S4-147' (pollen parent). Seeds of ‘Yotsuboshi’ were gathered in a naturally lit greenhouse at the Science and Technology Promotion Centre of Mie Prefectural Government, Japan. Strawberry seeds were sown on soil in plug trays and grown in an incubator at 25 °C, in a 16 h/8 h light/dark scheme at the photosynthetic photon flux density of 150 μmol m^−2^ s^−1^. Alternatively, strawberry seeds were surface-sterilized in a solution containing 0.01% Contaminon (a detergent; Fujifilm-Wako, Osaka, Japan) and 0.01% sodium hypochlorite for 5 min. Seeds were rinsed with sterilized distilled water three times, sown on half-strength MS medium solidified with 0.7% agar^48^, and then grown in the incubator. Cultivar ‘Dover’ was also grown on the same medium. One month after inoculation of viral RNA, strawberry plants in plug trays or medium were transferred to soil in plastic pots and grown in the incubator or in a naturally lit greenhouse.

### Construction of the ALSV vectors

Plasmid vectors pCALSR1 and pCALSR2, which encode the RNA1 and RNA2 genomes of ALSV, respectively, were used for the preparation of ALSV vectors^49^. The wtALSV vector (Fig. 1a) was prepared using pCALSR1 and pCALSR2 without any insertion sequence. For preparation of ALSV-FaPDS vector, a 201 nt fragment of strawberry *FaPDS* (FJ795342, nt 1469–1669) was artificially synthesized and introduced into the plasmid (Fasmac, Atsugi, Japan). This fragment was amplified with the Xho-FaPDS(+) primer (5ʹ-CCG CTC GAG GAT TCA GAA ATT GAT GCC-3ʹ) and BamH-FaPDS(−) primer (5ʹ-CGC GGA TCC GTC TCC AGT TAA ATA GAA ACC-3ʹ) to attach *Xho*I and *Bam*HI sites at either end of the fragment, using KOD Plus Neo DNA polymerase (Toyobo, Osaka, Japan). PCR was performed at 98 °C for 1 min, followed by 30 cycles of denaturation at 98 °C for 30 s, annealing at 55 °C for 30 s, and extension at 68 °C for 1 min. The reaction was finished with an additional extension at 68 °C for 3 min. This PCR product was introduced into the XSB site of pCALSR2 (RNA2 genome) after digestion with *Xho*I and *Bam*HI, so that the fragmental *PDS* codons were in frame with the codons of the RNA2 genome. For preparation of the ALSV-AtFT vector, the full-length *AtFT* coding sequence without the stop codon^34^ was introduced into the XSB site of pCALSR2 in-frame, after digestion with *Xho*I and *Bam*HI.

### Preparation of viral RNA

Viral RNA was prepared as previously described^48^. In summary, pCALSR1 and pCALSR2 plasmids were independently introduced into *A. tumefaciens* strain GV3101::pMP90. *Nicotiana benthamiana* leaves were inoculated by a 1:1:1 mixture of *Agrobacterium* culture transformed with pCALSR1, pCALSR2 or a transient expression plasmid encoding the HC-Pro silencing suppressor of clover yellow vein virus^36^. One month after inoculation, upper leaves were tested by RT-PCR for viral infection. Infected leaves were ground with buffer and rub inoculated to *Chenopodium quinoa* leaves. ALSV was concentrated by homogenization of infected *Chenopodium* leaves and treatment with bentonite solution. Viral RNA was obtained by using phenol–chloroform (1:1) extraction from this solution and precipitated with ethanol. RNA concentration was estimated by ultraviolet (UV) absorbance at 260 nm, and stored at –80 °C.

### Inoculation of viral RNA to strawberry

Gold particles (0.6 μm) (Bio-Rad Laboratories, Hercules, CA, USA) were coated with viral RNA as described previously^38^. For each bombardment of wtALSV, 7 μg of RNA was used, whereas 10 μg of RNA was used for bombardment of ALSV-FaPDS or ALSV-AtFT. Strawberry plants generated 3–4 true leaves at 1 month after sowing. The two expanded true leaves were inoculated by bombardment of RNA-coated gold particles by using a GDS-80 gene gun system (NepaGene, Ichikawa, Japan). Two shots of gold particles were used for each leaf, at the pressure of 20 psi. Gold particles are visible on leaves as brown coloration after successful bombardment.

### RNA extraction from strawberry

The procedure of total RNA extraction was slightly modified from a previous report^50^. Strawberry leaves (half of one leaf) were excised from the tip of the uppermost compound leaf and collected in 2 mL plastic tubes together with two stainless beads (SUB-50, φ 4.8 mm, Tomy, Tokyo, Japan), and frozen at –80 °C for at least 1 h. Leaves were crushed by using a MicroSmash MS-100R (Tomy) at 2500 rpm for 30 s. Tubes were briefly centrifuged, and 750 μL of RNA extraction buffer (0.1 M Tris-HCl, pH 8.0; 25 mM ethylenediaminetetraacetic acid, pH 8.0; 2 M NaCl; 2% cetyltrimethylammonium bromide, 2% polyvinylpyrrolidone; and 2% 2-mercaptoethanol) was added. Tubes were mixed again by using MicroSmash at 3000 rpm for 30 s, then incubated at 65 °C for 20 min, followed by the addition of 750 μL chloroform, vortexed for 2 min and centrifuged at 7827 × *g* for 10 min at 4 °C. A 720 μL fraction of the upper (water) phase was recovered to a new 1.5 mL tube, to which 240 μL LiCl (7.5 M) was added, mixed thoroughly and placed at –80 °C for at least 20 min. RNA was precipitated by centrifugation at 15,342 × *g* for 50 min at 4 °C, the supernatant was discarded, and the pellet rinsed with 1 mL of 80% ethanol and centrifuged at 15,342 × *g* for 5 min at 4 °C. The supernatant was discarded, the pellet was dried and sterilized distilled water was added to dissolve the pellet. RNA concentration was estimated by UV absorbance at 260 nm.

### Reverse transcription-polymerase chain reaction

Total RNA (1 μg) was reverse transcribed with oligo-(dT)_20_ primer, by using ReverTra Ace reverse transcriptase (Toyobo). The reaction was performed at 42 °C for 60 min, terminated at 99 °C for 5 min and held at 4 °C. Resultant cDNA (complementary DNA) was amplified by PCR as follows. The cDNA sample was amplified using ALSR2-999(+) (5ʹ-GCT CTC TGT AGT TAT TCT GCA G-3ʹ) and ALSR2-1437(−) (5ʹ-GAC CTT CTA GCA GAT TTG GG-3ʹ) primers with Ex Taq DNA polymerase (TaKaRa, Kusatsu, Japan). The reaction was performed at 95 °C for 1 min, followed by 40 cycles of denaturation at 94 °C for 20 s, annealing at 55 °C for 20 s and extension at 72 °C for 30 s. This PCR product was electrophoresed in a 1.5% agarose gel.

### Reverse transcription loop-mediated isothermal amplification

A protocol for detection of ALSV-RNA2 by RT-LAMP was recently developed for estimation of ALSV infection to gentian plants^43^. This protocol was followed to estimate ALSV infection to strawberry plants in the present study. Strawberry leaves were pricked with a wooden toothpick five times, and then the tip of the toothpick was rinsed in 100 μL extraction buffer (Tris-HCl, pH 8.0, 100 mM). Next, 2 μL of this solution was applied to a 25 μL total reaction volume. RT-LAMP reaction was performed by using the Loopamp RNA Amplification Kit (Eiken Chemical, Tokyo, Japan) at 63 °C for 60 min. The LAMP primers for ALSV detection were FIP (5ʹ-GAA GTG GCA CTC TTA GTT GGT AAA TTG ATT ACC TAA ATA GTT GCA ATG G-3ʹ), BIP (5ʹ-CAG GCC AGG TCA GGA TTT TGA CTA GGT GTA ACC AGC TTT G-3ʹ), F3 (5ʹ-GCA AAC AAT ACG TGG GAA G-3ʹ) and B3 (5ʹ-AGA GAA AAA GAA AAG GAC TCA A-3ʹ). Primer concentrations in the reaction were 1.5 μM for FIP and BIP, and 0.2 μM for F3 and B3. RT-LAMP amplification was detected by the green fluorescence of calcein (included in the Fluorescent Detection Reagent, Eiken Chemical) emitted when reaction tubes were directly illuminated by UV.

### In situ hybridization

The in situ hybridization analysis for detection of the RNA of ALSV in strawberry tissues was performed following a previously described protocol^44^. In summary, immature strawberry fruits (including sepals and receptacles) were fixed in FAA solution (formaldehyde alcohol acetic acid; formalin:ethanol:acetic acid:water = 10:50:5:35, v v^−1^), dehydrated by serial concentrations of ethanol and lemozol, and embedded in Paraplast Plus (Sigma-Aldrich, St. Louis, MO, USA). Sections prepared at 12 μm thickness were extended on APS-coated glass slides (Matsunami Glass, Osaka, Japan), deparaffinized and dehydrated. Slides were treated with proteinase K, re-fixed and a digoxigenin (DIG)-labelled probe that was complementary to the Vp24 region (nt 2619–3617) of the RNA2 of ALSV was hybridized; alternatively, a DIG-labelled probe complementary to the P1 region (nt 132–1045) of soybean mosaic virus was hybridized as a negative control. Slides were rinsed, then probes were labelled with sheep anti-DIG Conjugated Alkaline Phosphatase (Roche, Basel, Switzerland), rinsed again and stained with chromogenic substrate NBT/BCIP (Roche) to generate dark blue indigo and formazan dyes. The reaction was stopped by dipping the slide in water. Slides were dehydrated, dried and mounted in Entellan New (Merck, Darmstadt, Germany).
